# Implementation of a Laboratory-Integrated Clinical Decision Support System for CKD Screening and Progression Detection in Primary Care

**DOI:** 10.3390/biomedicines14091934

**Published:** 2026-08-28

**Authors:** Jordi Tortosa-Carreres, Jonnathan Acevedo-Galvis, Mónica Piqueras, William Harold, Carmen Ramos, Jose Luis Górriz, Enrique Rodríguez-Borja, Francisco Brotons-Muntó, Eugenia Avelino-Hidalgo, Pablo Molina, Begoña Laíz-Marro

**Affiliations:** 1 Laboratory Department, La Fe University and Polytechnic Hospital, Avinguda de Fernando Abril Martorell 106, Quatre Carreres, 46026 Valencia, Spain; acevedo_jongal@gva.es (J.A.-G.); laiz_beg@gva.es (B.L.-M.); 2 Clinical Laboratory Medicine Research Group, Health Research Institute Hospital La Fe (IISLaFe), 46026 Valencia, Spain; 3 Respiratory Diseases Research Group, Health Research Institute Hospital La Fe (IISLaFe), 46026 Valencia, Spain; 4 Nephrology Department, La Fe University and Polytechnic Hospital, Avinguda de Fernando Abril Martorell 106, Quatre Carreres, 46026 Valencia, Spain; rapley_wil@gva.es (W.H.); carmelaramos88@gmail.com (C.R.); pablo.molina-vila@uv.es (P.M.); 5 Metabolism in Renal Disease (MET-REN) Research Group, Health Research Institute Hospital La Fe (IISLaFe), 46026 Valencia, Spain; 6 Nephrology Department, University Clinical Hospital of Valencia, INCLIVA, Avenida de Blasco Ibáñez 17, 46010 Valencia, Spain; jlgorriz@gmail.com; 7 Department of Medicine, Universitat de València, 46010 Valencia, Spain; 8 Laboratory Department, University Clinical Hospital of Valencia, Avenida de Blasco Ibáñez 17, 46010 Valencia, Spain; rodriguez_enr@gva.es; 9 Family and Community Medicine Department, Trinitat I Primary Care Center, La Fe University and Polytechnic Hospital, 46026 Valencia, Spain; brotons_fra@gva.es; 10 Family and Community Medicine Department, Campanar II Primary Care Center, La Fe University and Polytechnic Hospital, 46026 Valencia, Spain; avelino_eug@gva.es

**Keywords:** kidney failure, chronic, decision support systems, clinical, laboratory information systems

## Abstract

**Background**: CKD remains substantially underdiagnosed and inadequately monitored. **Objectives**: To evaluate whether a laboratory-integrated CDSS enables opportunistic CKD detection and longitudinal surveillance in Primary Care via automated KDIGO stratification and progression detection. **Methods**: We implemented a CDSS embedded within the laboratory information system at Hospital Universitari i Politècnic La Fe, a tertiary university hospital in València, Spain. A one-time baseline profile screened patients with diabetes, hypertension, or age 60–80 years without documented advanced CKD, while a follow-up profile targeted patients with advanced CKD or those previously assessed by baseline screening. Both profiles applied KDIGO risk stratification and automated detection of renal and albuminuric progression based on predefined criteria, classifying each patient into one of three action categories: no CKD or low-risk requiring no action; CKD requiring Primary Care monitoring; or CKD meeting criteria for automated nephrology referral. Missed opportunities for albuminuria monitoring and economic impact of the intervention were evaluated. **Results**: During a 4-month period, the CDSS processed 10,733 requests, of which 10,414 were evaluable. No further action was required in 8661 patients (83.2%), monitoring was recommended in 1591 (15.3%), and nephrology referral was triggered in 162 (1.6%). Among 82 patients with albuminuric progression, 39% had not undergone ACR testing for more than two years despite ongoing Primary Care contact. Following implementation, ACR testing increased by 15.0% compared with the previous year, resulting in an additional analytical cost of EUR 1550.54. **Conclusions**: The CDSS enabled systematic CKD detection and surveillance in Primary Care, identifying silent renal and albuminuric progression and supporting nephrology referral at minimal cost.

## 1. Introduction

Chronic kidney disease (CKD), defined by structural or functional kidney abnormalities persisting over three months, affects an estimated 788 million adults worldwide and represents a major burden of morbidity, mortality, and healthcare cost [1,2,3]. In Spain, it affects 10–15% of adults (~5 million people), with rising prevalence and a substantial proportion remaining undiagnosed [4,5]. Economic impact is concentrated in advanced stages, with renal replacement therapy accounting for nearly half of CKD-related costs despite affecting few patients [6]. CKD is closely linked to diabetes mellitus (DM), arterial hypertension (HTN), and cardiovascular disease as both risk factors and consequences [7,8]; accordingly, the 2021 ESC guidelines recognize CKD as an independent cardiovascular risk factor, and KDIGO 2024 recommends risk stratification using estimated glomerular filtration rate (eGFR) and albumin-to-creatinine ratio (ACR) [9,10].

Despite the availability of simple laboratory markers, CKD remains underdiagnosed and insufficiently monitored. Although eGFR is routinely available, ACR testing is frequently omitted even in high-risk patients. Large real-world studies have shown that annual eGFR testing approaches 90% in adults with DM, whereas ACR testing is performed in only approximately half of patients; globally, ACR testing rates are as low as 35.1% in diabetes and 4.1% in HTN [11,12,13,14,15]. The reasons underlying this underuse are likely multifactorial and may include asymptomatic early disease, inconsistent implementation of guideline-recommended testing, and organizational barriers to systematic screening and longitudinal monitoring. This diagnostic gap is clinically relevant because albuminuria identifies patients at increased renal and cardiovascular risk before substantial loss of kidney function occurs and may facilitate earlier implementation of nephroprotective therapies [16,17].

Current guidelines support proactive CKD case finding in individuals at increased risk, including those with DM, HTN, or cardiovascular disease, while recommending periodic eGFR and ACR assessment for longitudinal monitoring in patients with established CKD [9,18]. In Spain, this is reinforced by the 2025–2028 National Chronicity Strategy consensus, which promotes systematic screening and integration of automated decision-support tools [19].

Clinical decision support systems (CDSSs) embedded within laboratory information systems can operationalize these recommendations by identifying eligible patients, adding omitted tests, and generating interpretative alerts [20,21]. Previous studies have demonstrated the feasibility of opportunistic albuminuria-based screening [22], but evidence remains limited regarding laboratory-integrated CDSSs supporting baseline CKD screening and longitudinal surveillance with automated detection of renal and albuminuric progression.

The aim of this study was to implement and evaluate a laboratory-integrated CDSS for opportunistic CKD screening and longitudinal surveillance in primary care, integrating KDIGO-based risk stratification with automated detection of renal and albuminuric progression. Specific objectives were to evaluate the clinical outputs generated by the system, characterize patients meeting automated nephrology referral criteria, quantify missed opportunities for albuminuria assessment, and estimate the operational impact of the intervention on laboratory activity.

## 2. Materials and Methods

The CDSS was implemented in the Clinical Laboratory Department of Hospital Universitari i Politècnic La Fe (València, Spain), a tertiary-care hospital whose laboratory also serves as the reference clinical laboratory for Primary Care centers within its healthcare department. Thus, although the CDSS infrastructure was centralized within a tertiary hospital laboratory, the target population and laboratory requests evaluated in this study originated specifically from Primary Care.

Automated rules were developed using AlinIQ^®^ Clinical Decision Support (Abbott Laboratories, Abbott Park, IL, USA), powered by RippleDown^®^ (Pacific Knowledge Systems, Sydney, Australia), and bidirectionally integrated with the laboratory information system (LIS) (GestLab^®^, Clinisys, Chertsey, UK) and the corporate electronic health record. The platform provides a configurable rule engine with predefined functions for creating derived variables within the CDSS and executing rule-based actions within the LIS, whereas the CKD-specific decision logic and its configuration were entirely designed by the study team. AlinIQ^®^ automatically retrieved the complete laboratory history available in the LIS, together with demographic data and information on diagnoses and treatments. Historical measurements originated from routine physician-requested testing. For longitudinal surveillance, the predefined progression algorithms were applied using the most recent previous result meeting the corresponding temporal criteria.

### 2.1. Algorithm Design and Analytical Studies

The algorithm was based on the KDIGO G/A risk classification for CKD staging and prognosis and on the Spanish consensus document for CKD detection and management [4]. Its development built upon prior experience with CDSS-based CKD screening at Hospital Clínic Universitari (València) and was subsequently adapted to the clinical workflow, information systems, and clinical–analytical objectives of our institution.

Two automated laboratory profiles were developed for CKD detection and monitoring and embedded into the routine clinical laboratory workflow. Eligible patients were automatically identified using predefined inclusion criteria retrieved from the corporate electronic health record, while laboratory data were retrieved from the LIS.

**Figure 1:**
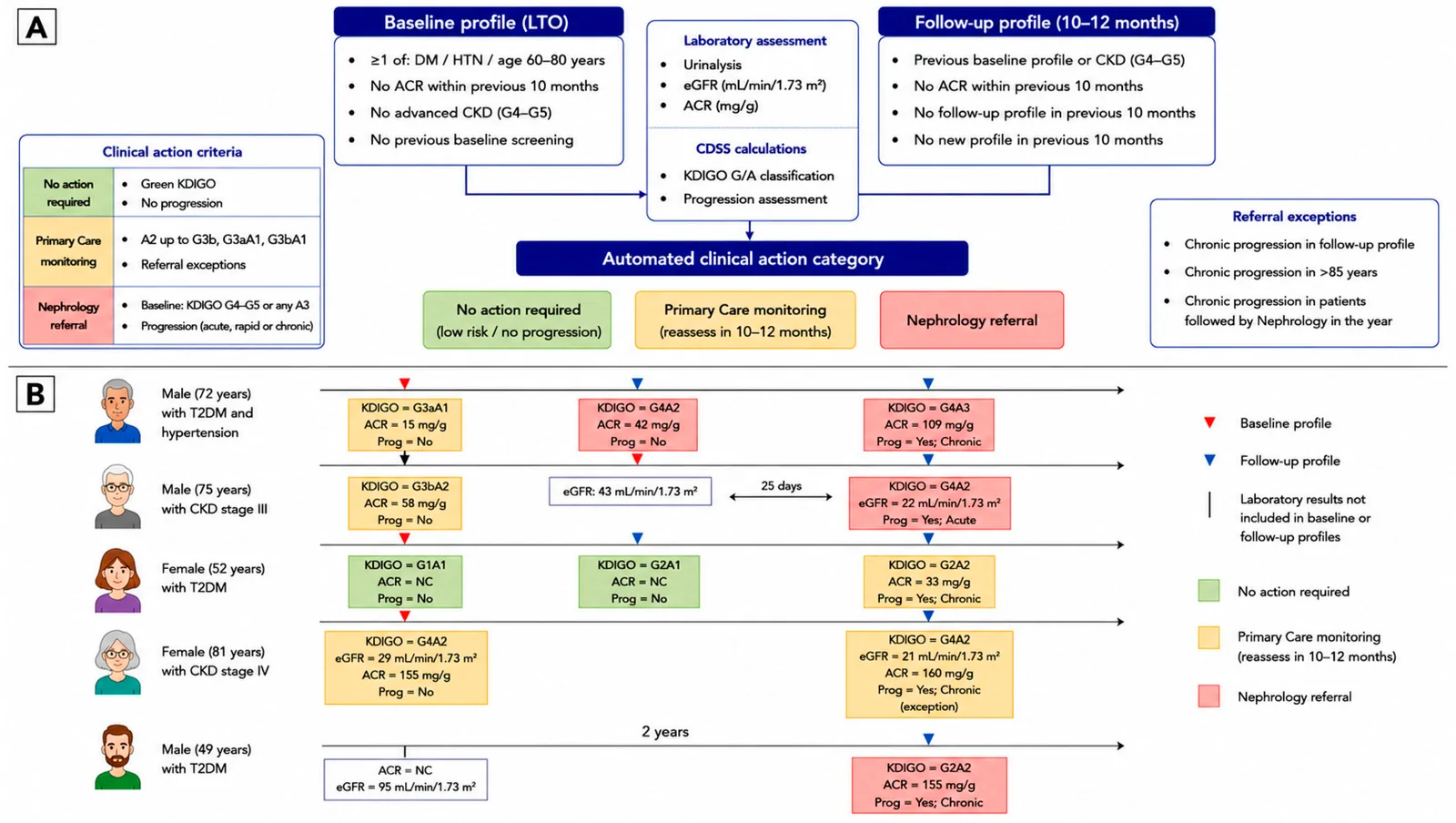
CDSS workflow and illustrative clinical scenarios. (**A**) Workflow of the automated CKD screening and follow-up strategy. (**B**) Representative clinical scenarios illustrating the CDSS decision logic. From top to bottom: (1) baseline screening identifying advanced CKD (G3aA1→G4A2) and subsequent chronic progression prompting Nephrology referral; (2) acute renal progression detected during follow-up leading to immediate Nephrology referral; (3) transition from no CKD to moderate-risk CKD with chronic progression requiring Primary Care monitoring; (4) chronic progression in a patient with advanced CKD managed as a referral exception because of age (>85 years); and (5) de novo detection of albuminuric progression during baseline screening. ACR, albumin-to-creatinine ratio; CDSS, clinical decision support system; CKD, chronic kidney disease; DM, diabetes mellitus; eGFR, estimated glomerular filtration rate; HTN, hypertension; LTO, once-in-a-lifetime; T2DM, type 2 diabetes mellitus.

The baseline profile was designed as a once-in-a-lifetime opportunistic screening strategy. Eligible patients had at least one of the following cardiorenal risk factors: DM, identified using active International Classification of Diseases, 10th Revision (ICD-10) codes E10–E11; HTN, identified using active ICD-10 codes I10, I15, I16 and I19; or age between 60 and 80 years. Patients with known advanced CKD (KDIGO stage ≥ G4 or ICD-10 codes N18.4–N18.6), a previous baseline or follow-up renal study, or albuminuria assessment during the preceding 10 months were excluded (Figure 1A).

The follow-up profile was applied to patients with known advanced CKD (ICD-10 codes N18.4–N18.6) or a previous baseline profile, provided that no baseline renal study or albuminuria assessment had been performed during the preceding 10 months. Both profiles included serum creatinine measurement, automatic eGFR calculation using the CKD-EPI equation [23], ACR, automated KDIGO risk classification [24], and longitudinal assessment of renal progression using previous laboratory results (Figure 1A). Figure 1B illustrates representative clinical scenarios, including baseline study activation, re-entry into follow-up, and automated report categories generated by the system.

### 2.2. Definition of Renal Progression

The algorithm incorporated three renal progression criteria based on the Spanish consensus document for CKD detection and management [4], subsequently adapted to the automated laboratory workflow: acute progression (AP), rapid progression (RP), and chronic progression (CP).

AP was defined by either a ≥30% increase in serum creatinine within one month in patients with baseline creatinine > 1.5 mg/dL, or a ≥30% decrease in eGFR over the same time interval.

RP was defined according to annual eGFR decline, either as a decrease of ≥15 mL/min/1.73 m^2^ per year in patients with eGFR < 50 mL/min/1.73 m^2^, or as a ≥25% annual reduction in eGFR in patients with eGFR < 40 mL/min/1.73 m^2^.

CP included three criteria: a decrease of ≥5 mL/min/1.73 m^2^ in eGFR over one year in patients with eGFR < 40 mL/min/1.73 m^2^, using a higher threshold of eGFR < 30 mL/min/1.73 m^2^ in patients aged 85 years or older; CP-ACR20, defined as a progressive ≥20% increase in ACR across each of the three previous consecutive measurements; and CP-ACR50, defined as a ≥50% increase in ACR compared with the previous measurement when the resulting ACR was >150 mg/g, using a higher threshold of >300 mg/g in patients aged 85 years or older.

### 2.3. Stratification, Referral Rules and Automated Reports

**Figure 2:**
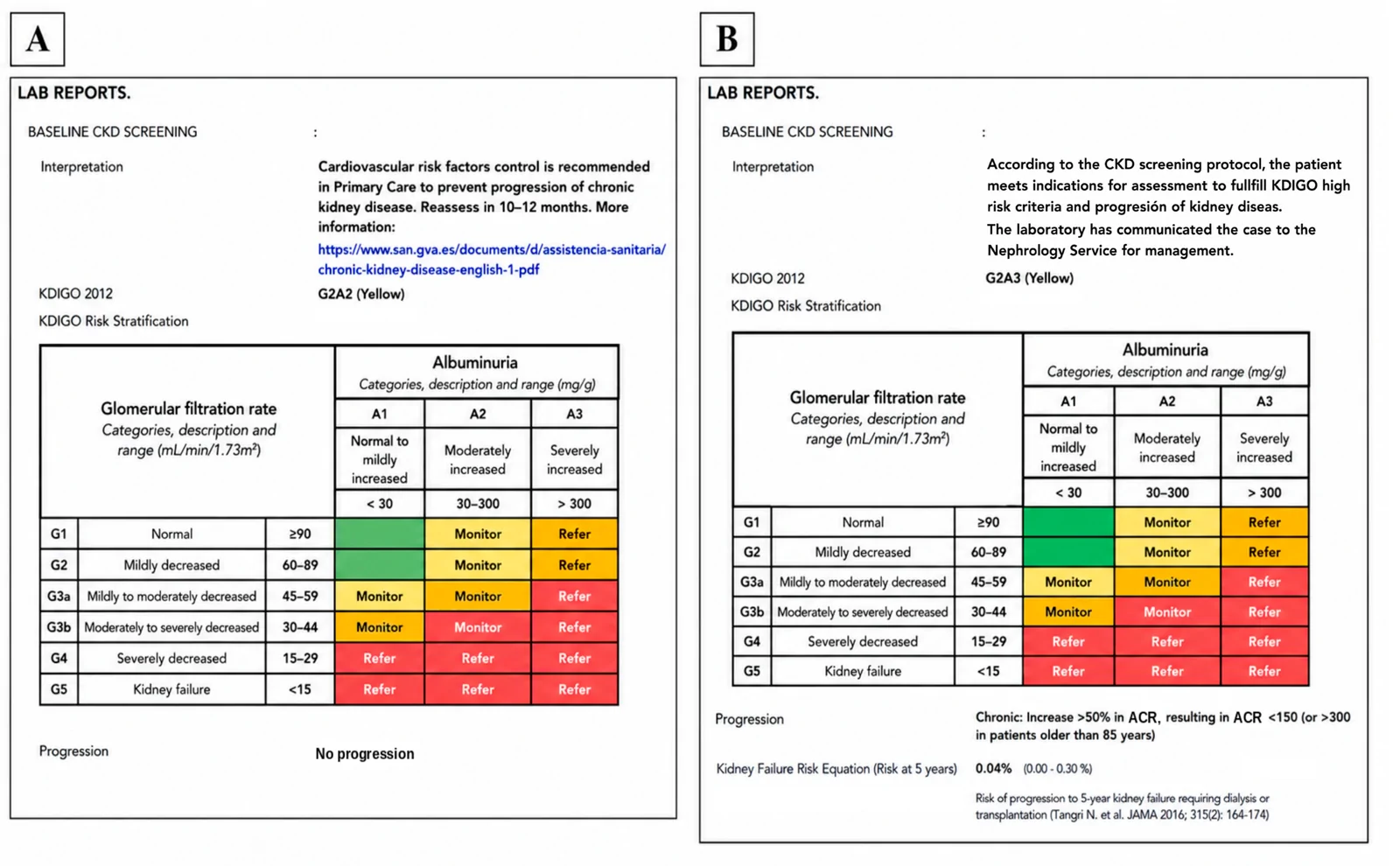
Examples of automated interpretative laboratory reports generated by the CDSS. (**A**) shows an intermediate-risk report without progression, including KDIGO-based classification and a recommendation for primary care monitoring. (**B**) shows a high-risk report with albuminuric progression, an automated recommendation for nephrology referral, and 5-year kidney failure risk estimated using the KFRE [25]. The colour-coded KDIGO matrix embedded in the report facilitates immediate clinical interpretation of kidney risk and the recommended clinical action. Reports were originally generated in Spanish and are presented here as English translations.

The CDSS assigned each patient to one of three predefined clinical action categories based on KDIGO G/A category (Figure 2) and renal progression status:

1. No action required: Patients assigned to this category were classified within the green KDIGO categories (G1A1 ang G2A1) and showed no evidence of renal progression.
2. Primary Care monitoring: Patients assigned to this category included those with KDIGO categories designated for monitoring by the study protocol (A2 categories up to G3b, together with G3aA1 and G3bA1), as well as patients meeting predefined referral exceptions: chronic progression (CP) detected exclusively during follow-up, CP in patients older than 85 years, and CP in patients who had already been followed by Nephrology during the previous year. In these situations, the CDSS generated an interpretative report providing a general recommendation for monitoring and optimization of cardiorenal risk factors in Primary Care, together with reassessment after 10–12 months and a link to the regional CKD clinical practice guideline of the Generalitat Valenciana (https://www.san.gva.es/documents/d/assistencia-sanitaria/enfermedad-renal-cronica-castellano-1-pdf, accessed on 14 December 2025).
3. Nephrology referral: Patients assigned to this category included those with KDIGO G4–G5 categories, any A3 albuminuria category, or any renal progression criterion detected during baseline screening. During follow-up, only patients with AP or RP were referred. For all patients in this category, the 5-year risk of kidney failure was automatically calculated using the 4-variable Kidney Failure Risk Equation (KFRE) [25].

For patients triggering an automated output, the CDSS generated a personalized interpretative laboratory report. This report included the assigned KDIGO G/A category, the corresponding KDIGO risk stratum, the clinical action level, and the KDIGO risk matrix to facilitate clinical interpretation. When renal progression was detected, the specific progression criterion was reported according to the predefined nomenclature (Figure 2). When criteria from different progression categories coexisted, the reported category was hierarchically assigned according to clinical urgency, prioritizing AP over RP and RP over CP. When multiple criteria within the same category were simultaneously fulfilled, a single criterion was reported to avoid redundant alerts and excessive report complexity.

### 2.4. ACR Determination and Analytical Considerations

In our setting, initial screening for albuminuria/proteinuria was performed using automated urine dipstick analysis on UF^®^ analyzers (Sysmex, Kobe, Japan) as a semiquantitative screening test. Serum creatinine, urinary creatinine, and urinary microalbumin were measured on Alinity c^®^ analyzers (Abbott Laboratories, Abbott Park, IL, USA). Quantitative measurement of urinary albumin and creatinine was performed at the same visit, using the same urine sample, only after a positive screening result [26].

For KDIGO classification purposes, samples with negative screening were automatically classified as A1, as were samples with quantitative ACR < 30 mg/g, urinary albumin below the analytical limit of detection (<0.1 mg/dL), or non-quantifiable results.

In addition, to preserve longitudinal data continuity and enable dynamic computational analysis of albuminuria trajectories, samples with negative screening were assigned an operational ACR value of 2.9 mg/g. This value was selected because it was immediately below the lowest ACR observed in a local cohort of 58 samples with negative screening that were subsequently quantified, in which the minimum value was 3 mg/g.

### 2.5. Data Analysis and Outcome Assessment

#### 2.5.1. Descriptive Analysis of CDSS Activity

Quantitative variables were expressed as median and interquartile range, and categorical variables as absolute frequencies and percentages. No inferential analyses or causal models were performed, given the observational, retrospective, and uncontrolled nature of the implementation.

The total number of requests processed by the system was described, distinguishing between baseline and follow-up profiles. Interpretable requests were classified according to the clinical action level generated by the algorithm, as previously described (no further action, monitoring, or nephrology referral). Non-classifiable requests were described separately.

Among requests requiring monitoring, predefined exceptions, high-risk KDIGO categories detected in follow-up studies, and intermediate KDIGO categories were described separately.

Among patients referred to Nephrology, demographic, clinical, analytical, and treatment-related characteristics were described overall and separately for baseline and follow-up studies. These variables included age, sex, eGFR, serum creatinine, ACR categories, hematuria, leukocyturia, bacteriuria, KFRE, treatment with renin–angiotensin system inhibitors (RASi), SGLT2 inhibitors, statins, and diuretics—classified as high-ceiling diuretics, low-ceiling diuretics, and potassium-sparing diuretics according to the Anatomical Therapeutic Chemical classification system—as well as the presence of DM and HTN. Reasons for Nephrology referral were categorized as high-/very-high-risk KDIGO classification, renal progression, or both. In patients referred because of renal progression, the frequency of acute, rapid, and chronic progression was described, as well as its distribution according to analytical profile type.

Statistical analyses were performed using R version 4.5.0 (R Foundation for Statistical Computing, Vienna, Austria).

#### 2.5.2. Chronic Progression Substudy

For the albuminuric chronic progression substudy, patients were included if they met two criteria: CP-ACR20 or CP-ACR50 progression and Nephrology referral criteria.

The time elapsed since the previous ACR measurement and whether patients underwent interim primary care laboratory testing with or without urine sample submission were evaluated as indicators of missed opportunities for earlier detection of albuminuric progression.

#### 2.5.3. Estimation of Economic Impact

The economic impact was estimated descriptively based on the actual analytical cost generated during the post-implementation period. For each request processed by the system, the determinations actually performed were identified, as not all samples required urinary sediment analysis or quantitative urinary albumin and creatinine measurement following a negative initial dipstick.

The unit costs considered were EUR 0.03 for serum creatinine, EUR 0.03 for urinary creatinine, EUR 0.21 for automated urine dipstick screening, EUR 0.75 for urinary sediment analysis, and EUR 1.50 for urinary microalbumin. Based on these unit costs, the total observed analytical cost during the study period was calculated.

To estimate the incremental cost potentially attributable to CDSS implementation, the additional increase in ACR requests relative to the recent baseline trend was applied to the total observed analytical cost. This additional increase was calculated as the difference between the relative increase observed from 2025 to 2026 and that observed from 2024 to 2025. The estimated incremental cost was obtained by applying this additional percentage increase to the total observed analytical cost.

#### 2.5.4. Declaration of Generative AI Use

Artificial intelligence tools (ChatGPT, GPT-5.6 Sol, OpenAI, San Francisco, CA, USA) were employed to improve language clarity and assist with grammatical editing. The authors take full responsibility for the subsequent revisions and for the final content of the published work.

## 3. Results

**Figure 3:**
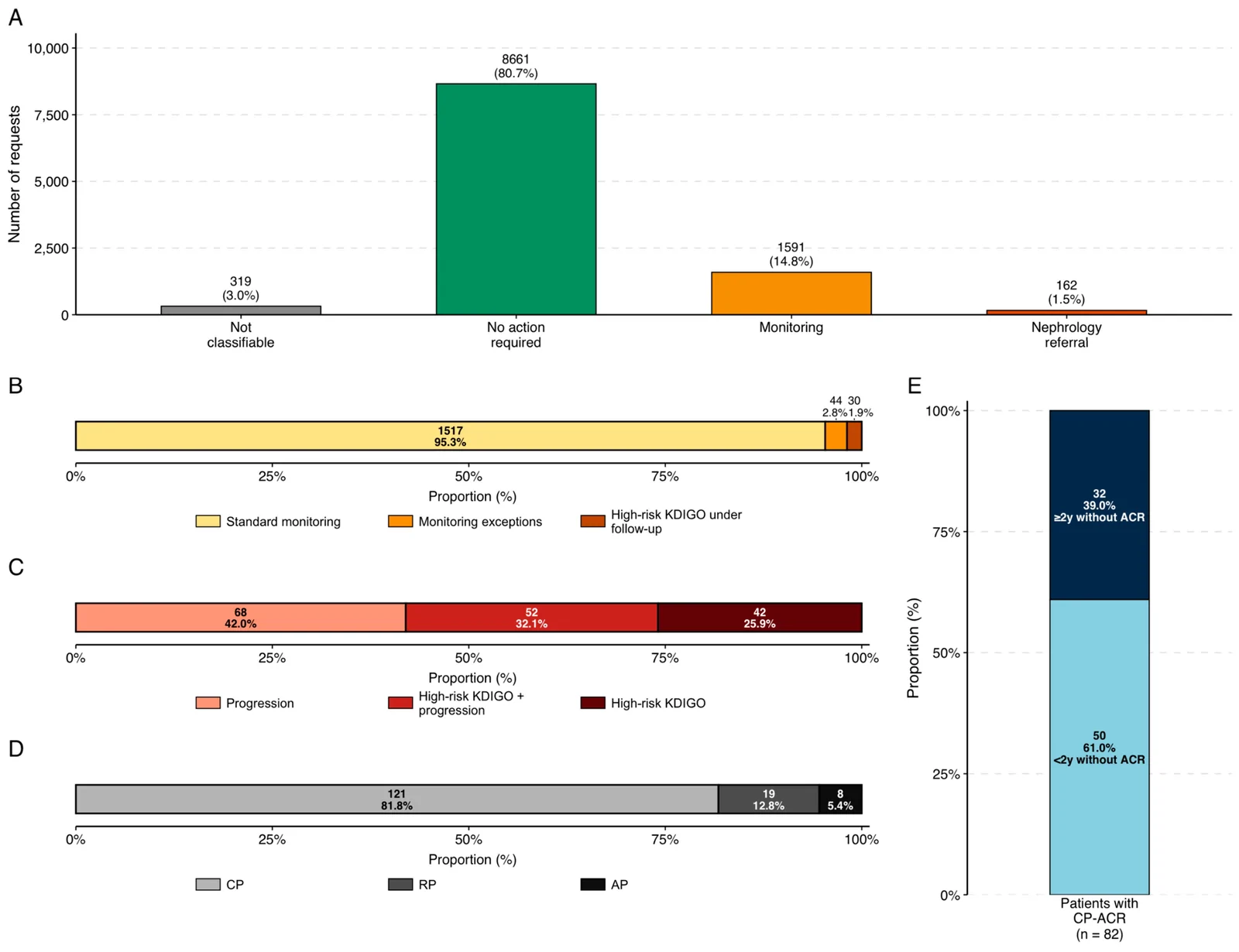
CDSS-generated clinical outputs, renal progression patterns, and missed opportunities for albuminuria monitoring. (**A**) shows the overall classification of processed requests according to the generated action level. “Not classifiable” refers to requests in which no complete clinical–analytical classification could be issued, mainly because no urine sample was submitted and no eGFR/creatinine-based progression criterion was detected. (**B**) shows the distribution of requests requiring monitoring, distinguishing standard monitoring alerts, monitoring exceptions, and high-risk KDIGO categories under follow-up. (**C**) shows the reasons for Nephrology referral. (**D**) shows the distribution of renal progression phenotypes detected by the system. (**E**) shows the proportion of patients included in the albuminuric progression substudy who had not undergone ACR assessment for at least two years despite ongoing contact with Primary Care. ACR, albumin-to-creatinine ratio; AP, acute progression; CP, chronic progression; CP-ACR, chronic albuminuric progression; KDIGO, Kidney Disease: Improving Global Outcomes; RP, rapid progression.

During the study period, the CDSS processed 10,733 requests. Overall, 2723 requests (25.4%) corresponded to patients with HTN and 1473 (13.7%) to patients with diabetes. Both risk factors were more frequent in the follow-up profile than in the baseline screening profile: HTN, 63.2% vs. 25.1%; diabetes, 36.8% vs. 13.6%. In 319 cases (3.0%), a definitive classification could not be issued due to unavailable samples or missing laboratory data. Among the remaining 10,414 interpretable requests, 8661 (83.2%) required no further action, 1591 (15.3%) required monitoring, and 162 (1.6%) met Nephrology referral criteria (Figure 3A). Detailed KDIGO categories stratified by profile type are shown in Table 1, while Table 2 summarizes the clinical, renal, and treatment characteristics of patients meeting nephrology referral criteria.

Among requests requiring monitoring, most were classified as yellow alerts (1517/1591; 95.3%), whereas predefined exception rules accounted for 44 cases (2.8%) and red KDIGO categories under follow-up accounted for 30 cases (1.9%) (Figure 3B).

Referral criteria were met in 162 requests: 68 (42.0%) were triggered by progression criteria alone, 52 (32.1%) by the combination of high-risk KDIGO classification and progression criteria, and 42 (25.9%) by high-risk KDIGO classification alone (Figure 3C).

Progression criteria were identified in 148 requests (1.4% of all processed requests). The majority corresponded to chronic progression (*n* = 121; 81.8%), followed by rapid progression (*n* = 19; 12.8%) and acute progression (*n* = 8; 5.4%). Progression was more frequent in the follow-up profile than in the baseline screening profile (19.1% vs. 1.3%) (Figure 3D).

Among progression-related referrals, chronic progression was the predominant pattern (*n* = 92; 78.0%), followed by rapid progression (*n* = 19; 16.1%) and acute progression (*n* = 7; 5.9%). Most progression-related referrals occurred during the baseline screening profile (114/118), whereas 4 occurred in the follow-up profile.

The chronic progression substudy included 82 eligible patients. Thirty-two patients (39.0%) had not undergone ACR measurement for at least two years, despite 29 of them (90.6%) showing microalbuminuria at the index assessment. During this interval, 25 patients had undergone primary care blood testing, and 14 had also submitted a urine sample, representing missed opportunities for earlier albuminuria assessment (Figure 3E).

**Figure 4:**
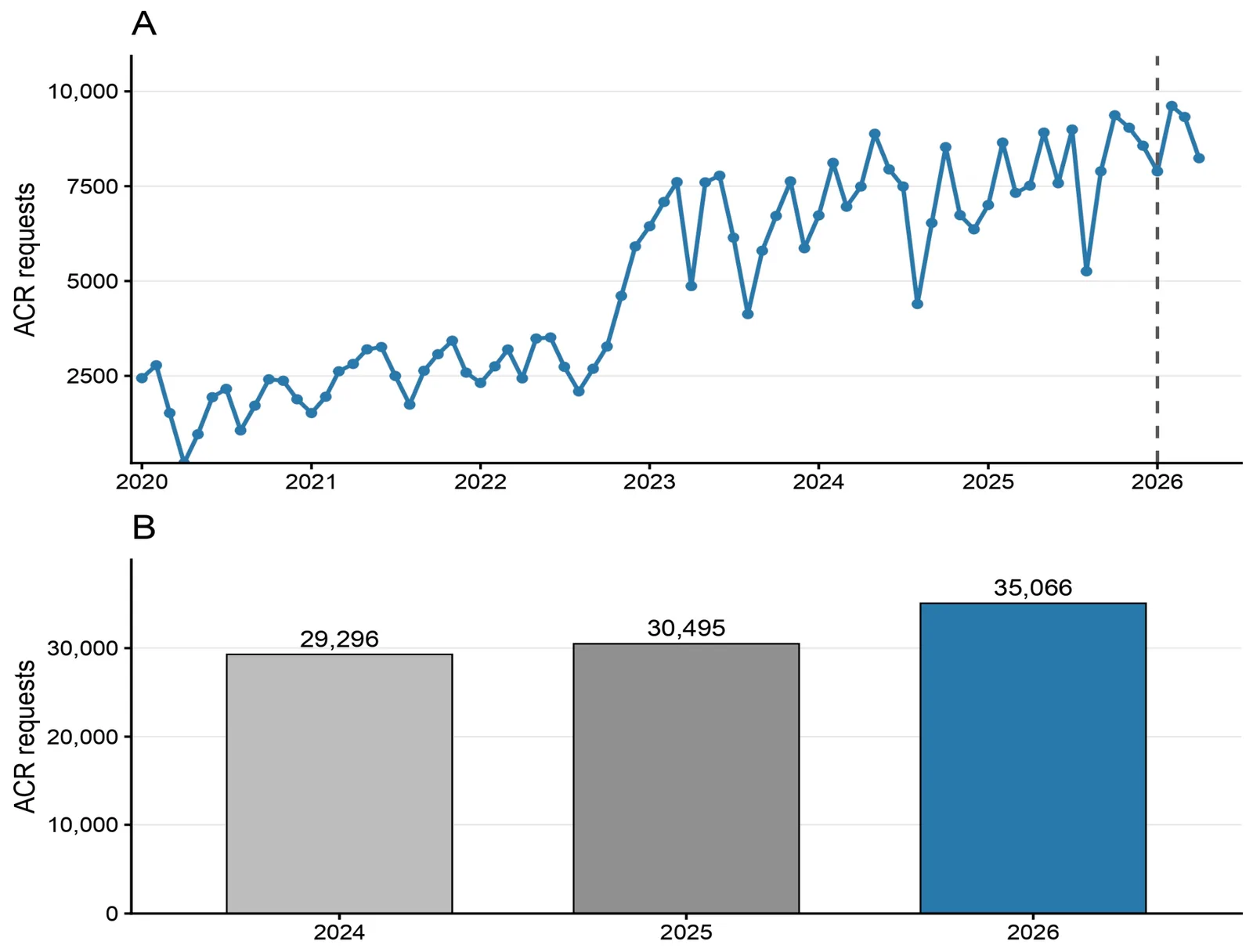
Temporal trends in ACR requests. (**A**) shows the monthly number of ACR requests from 2022 to 2026. The dashed grey line indicates implementation of the automated CDSS strategy in January 2026. (**B**) compares the total number of ACR requests performed between January and April in 2024, 2025, and 2026, illustrating the recent increase in activity after implementation. ACR, albumin-to-creatinine ratio.

Between January and April 2026, 35,066 urinary ACR determinations were performed, compared with 30,495 during the same period in 2025, representing a 15.0% relative increase. By contrast, the increase observed between 2024 and 2025 was 4.1% (29,296 vs. 30,495 determinations) (Figure 4). During the post-implementation period, after excluding requests cancelled because no urine sample was submitted, the total observed analytical cost was EUR 14,225.22. Applying the additional relative increase in ACR requests observed after implementation (10.9%), the estimated additional analytical cost associated with CDSS implementation was EUR 1550.54.

## 4. Discussion

This study describes the implementation of a laboratory-integrated CDSS designed to support opportunistic CKD screening and longitudinal surveillance in Primary Care. The principal finding was that most clinically actionable cases identified by the system were associated with renal progression rather than with baseline CKD classification alone. More than two thirds of nephrology referrals were triggered either exclusively by progression criteria or by the combination of progression and high-risk KDIGO categories, suggesting that longitudinal surveillance complements conventional cross-sectional assessment by providing additional clinically relevant information that would otherwise not be captured. Importantly, only 1.6% of processed requests generated a nephrology referral despite systematic population-based surveillance, indicating that the algorithm generated a limited number of specialist referrals while identifying patients with clinically silent disease progression. Current CKD management is largely based on the integration of kidney function and albuminuria according to KDIGO recommendations [8]. While this approach provides robust prognostic stratification, our findings indicate that patients within the same KDIGO category may follow different clinical trajectories depending on the stability or progression of their renal parameters. The predominance of progression-related referrals observed in our cohort suggests that automated longitudinal surveillance may help identify patients with active CKD progression who might not be recognized through isolated cross-sectional assessments. In this regard, our results suggest that longitudinal monitoring should complement, rather than replace, conventional risk stratification. Another important finding was the predominance of chronic progression, particularly progression driven by worsening albuminuria. This observation reinforces previous reports showing persistent underutilization of ACR testing despite its recognized prognostic value and its central role in CKD classification and management [6,8,9,10,11,27]. However, our results suggest that the challenge extends beyond initial CKD screening. A substantial proportion of patients with clinically relevant albuminuric progression had not undergone ACR assessment for prolonged periods despite maintaining regular contact with healthcare services.

The chronic progression substudy provides further insight into this issue. Nearly 40% of patients with albuminuric progression had not undergone ACR assessments for more than two years despite undergoing interim laboratory evaluations and, in some cases, providing urine samples during this period. These findings highlight missed opportunities for earlier identification of disease progression and suggest that current deficiencies in CKD surveillance may arise not only from under-screening but also from the absence of mechanisms ensuring systematic longitudinal monitoring. Importantly, they also indicate that inadequate albuminuria surveillance may occur even in patients already engaged with the healthcare system, highlighting a potential gap between guideline recommendations and routine clinical practice. This observation also underscores the importance of ACR availability as a decision variable within the CDSS, as it contributes both to KDIGO risk stratification and to the detection of albuminuric progression.

Previous CDSS-based initiatives have demonstrated the feasibility of automated CKD screening, KDIGO classification, and nephrology referral support in at-risk populations [22]. Similarly, albuminuria-based screening strategies have shown the ability to identify previously unrecognized CKD among high-risk individuals [27,28]. However, most published approaches have focused primarily on the detection of prevalent disease. The present system extends this concept by incorporating automated progression detection into routine laboratory workflows, allowing identification not only of patients with previously unrecognized CKD but also of those experiencing clinically silent deterioration despite ongoing healthcare contact. This distinction may be particularly relevant given the substantial burden of undiagnosed CKD reported internationally, where a large proportion of patients fulfilling laboratory criteria for CKD remain uncoded and unrecognized despite regular interaction with healthcare services [29,30].

From a methodological perspective, a distinctive feature of the proposed strategy was the pragmatic assignment of ACR values to screening-negative samples. Although these values do not represent true quantitative measurements, they enabled preservation of longitudinal albuminuria trajectories in a setting where quantitative ACR determination relies on reflex testing following an initial positive screening result. This approach may facilitate the implementation of longitudinal surveillance algorithms in laboratories using similar analytical workflows, although further validation is warranted. However, future applications involving inferential analyses or more complex statistical models may require alternative approaches for handling non-quantified ACR values.

From an operational perspective, the strategy proved feasible at scale within our institution. More than 10,000 requests were evaluated during the study period, while only a small proportion generated specialist referral. The tiered intervention model enabled prioritization of nephrology resources while providing structured recommendations for patients suitable for follow-up within Primary Care.

Although formal cost-effectiveness analyses were beyond the scope of this study, these findings are consistent with previous evidence suggesting that albuminuria-based detection and monitoring strategies may become increasingly valuable in the era of modern nephroprotective therapies, including SGLT2 inhibitors [31].

Several limitations should be acknowledged. First, the observational and uncontrolled design precludes conclusions regarding causal effects on clinical outcomes. Second, it was not possible to determine which ACR measurements would have been requested independently of the CDSS, limiting the accuracy of the estimated incremental activity and associated costs. Third, the economic evaluation was restricted to direct analytical costs and did not assess potential downstream effects including treatment optimization, prevention of kidney failure, cardiovascular outcomes, or healthcare utilization. Fourth, the study was conducted within a single healthcare department using local workflows and information systems, which may limit its generalizability. Fifth, albuminuria classification relied on an initial dipstick screening strategy that may underestimate low-grade albuminuria. Sixth, given the Primary Care setting and the population-scale nature of the strategy, progression assessment relied on serum creatinine/eGFR and ACR, as widely available, low-cost biomarkers recommended by clinical guidelines [4], without incorporating additional biomarkers such as cystatin C or suPAR. Seventh, clinician adherence to CDSS recommendations and their impact on therapeutic decisions, referral completion, and long-term renal or cardiovascular outcomes were not evaluated. Eighth, the initial implementation was intentionally restricted to selected high-risk groups (DM, HTN, and adults aged 60–80 years) to maintain operational feasibility and avoid excessive increases in ACR testing and nephrology referrals. Finally, implementation relied on an established laboratory information infrastructure and a dedicated CDSS platform, which may limit direct transferability to smaller or less digitally integrated laboratory settings.

Future developments of the CDSS may include extending this strategy to younger adults with obesity, a family history of early-onset CKD, heart failure, or established cardiovascular disease who are not currently included in the screening criteria.

## 5. Conclusions

The implementation of a laboratory-integrated CDSS enabled opportunistic CKD screening and longitudinal surveillance in routine clinical practice. The system identified clinically silent renal and albuminuric progression, uncovered missed opportunities for guideline-recommended monitoring, and generated clinically actionable information with minimal additional analytical costs. These findings support the use of a laboratory-integrated CDSS as a scalable and resource-efficient strategy to strengthen CKD detection and longitudinal surveillance, while facilitating risk-based management in high-risk populations.

## Figures and Tables

**Table 1:** KDIGO category distribution in baseline screening and follow-up profiles.

KDIGO Category	Overall, *n* (%)	Baseline Screening, *n* (%)	Follow-Up, *n* (%)
G1A1	4281 (41.1%)	4281 (41.3%)	0 (0.0%)
G1A2	240 (2.3%)	240 (2.3%)	0 (0.0%)
G1A3	13 (0.1%)	13 (0.1%)	0 (0.0%)
G2A1	4383 (42.1%)	4380 (42.2%)	3 (4.8%)
G2A2	416 (4.0%)	414 (4.0%)	2 (3.2%)
G2A3	47 (0.5%)	47 (0.5%)	0 (0.0%)
G3aA1	543 (5.2%)	539 (5.2%)	4 (6.3%)
G3aA2	130 (1.2%)	127 (1.2%)	3 (4.8%)
G3aA3	19 (0.2%)	18 (0.2%)	1 (1.6%)
G3bA1	164 (1.6%)	156 (1.5%)	8 (12.7%)
G3bA2	85 (0.8%)	79 (0.8%)	6 (9.5%)
G3bA3	25 (0.2%)	23 (0.2%)	2 (3.2%)
G4A1	27 (0.3%)	16 (0.2%)	11 (17.5%)
G4A2	22 (0.2%)	13 (0.1%)	9 (14.3%)
G4A3	13 (0.1%)	5 (0.05%)	8 (12.7%)
G5A2	3 (0.03%)	0 (0.0%)	3 (4.8%)
G5A3	3 (0.03%)	0 (0.0%)	3 (4.8%)
Total	10,414 (100.0%)	10,351 (100.0%)	63 (100.0%)

**Table 2:** Clinical, renal, and treatment characteristics of patients assessed for nephrology referral.

	Overall (*n* = 162)	Baseline (*n* = 154)	Follow-Up (*n* = 8)
Male sex	91 (56.2%)	88 (57.1%)	3 (37.5%)
Age, years	75 (65–81)	75 (65–80.8)	81.5 (76.2–86)
eGFR, mL/min/1.73 m^2^	58.5 (35–84)	60 (36–84)	19 (18.5–28.5)
Creatinine, mg/dL	1.2 (0.8–1.6)	1.1 (0.8–1.5)	2.2 (1.9–2.7)
Negative dipstick screening *	11 (7.0%)	11 (7.2%)	0 (0.0%)
ACR < 30 mg/g *	26 (16.5%)	25 (16.4%)	1 (16.7%)
ACR 30–300 mg/g §	60 (38.0%)	56 (36.8%)	4 (66.7%)
ACR > 300 mg/g §	72 (45.6%)	71 (46.7%)	1 (16.7%)
Urine not submitted †	4 (2.5%)	2 (1.3%)	2 (25.0%)
Hematuria ‡§	55 (34.8%)	53 (34.9%)	2 (33.3%)
Leukocyturia ≥ 15/µL §	60 (38.0%)	57 (37.5%)	3 (50.0%)
Bacteriuria ≥ 1000/µL §	29 (18.4%)	27 (17.8%)	2 (33.3%)
KFRE ≥ 3% ¶ (25)	42 (28.4%)	36 (25.4%)	6 (100.0%)
KFRE, %	0.6 (0–3.7)	0.5 (0–3)	18.7 (8–40.7)
SGLT2 inhibitor treatment	43 (26.5%)	39 (25.3%)	4 (50.0%)
High-ceiling diuretics	32 (19.8%)	26 (16.9%)	6 (75.0%)
Low-ceiling diuretics	7 (4.3%)	6 (3.9%)	1 (12.5%)
Potassium-sparing diuretics	11 (6.8%)	10 (6.5%)	1 (12.5%)
RASi	115 (71.4%)	114 (74.5%)	1 (12.5%)
Statin treatment	98 (60.5%)	93 (60.4%)	5 (62.5%)
DM	67 (41.4%)	65 (42.2%)	2 (25.0%)
HTN	94 (58.0%)	90 (58.4%)	4 (50.0%)

Data are shown as *n* (%) for categorical variables and median (IQR) for continuous variables. ACR, albumin-to-creatinine ratio; DM, diabetes mellitus; eGFR, estimated glomerular filtration rate; HTN, hypertension; KFRE, Kidney Failure Risk Equation; RASi, Renin–Angiotensin System inhibitors; SGLT2, sodium–glucose cotransporter 2. * Negative urine dipstick screening indicates samples with a negative urine dipstick result for which quantitative ACR measurement was not performed. For descriptive purposes, these samples were classified as ACR < 30 mg/g according to the study algorithm. † Percentages for “urine not submitted” were calculated using the total number of patients in each group as the denominator. These patients were still assessed for nephrology referral because referral eligibility could also be triggered by renal function criteria, including reduced eGFR or evidence of eGFR/creatinine-based progression, even in the absence of a valid urine sample. ‡ Hematuria was defined as >15 erythrocytes/µL in men and >25 erythrocytes/µL in women. § Percentages for ACR categories and urinary sediment abnormalities were calculated among patients with available urine assessment. Negative dipstick-based screening was considered negative for urinary sediment abnormalities. ¶ Percentages for KFRE ≥ 3% were calculated among patients with available KFRE.

## Data Availability

The data supporting the findings of this study are available from the corresponding authors upon reasonable request.

## Abbreviations

The following abbreviations are used in this manuscript:

ACR	urine albumin-to-creatinine ratio
AP	acute progression
CDSS	clinical decision support system
CKD	chronic kidney disease
CP	chronic progression
CP-ACR20	chronic progression defined as a progressive ≥20% increase in ACR across each of the three previous consecutive measurements
CP-ACR50	chronic progression defined as a ≥50% increase in ACR compared with the previous measurement when the resulting was >150 mg/g (or >300 mg/g in patients aged ≥85 years)
DM	diabetes mellitus
eGFR	estimated glomerular filtration rate
HTN	arterial hypertension
ICD-10	International Classification of Diseases, 10th Revision
KDIGO	Kidney Disease: Improving Global Outcomes
KFRE	Kidney Failure Risk Equation
LIS	laboratory information system
RASi	treatment with renin–angiotensin system inhibitors
RP	rapid progression
